# Expanding the application of tryptophan: Industrial biomanufacturing of tryptophan derivatives

**DOI:** 10.3389/fmicb.2023.1099098

**Published:** 2023-03-23

**Authors:** Shujian Xiao, Zhen Wang, Bangxu Wang, Bo Hou, Jie Cheng, Ting Bai, Yin Zhang, Wei Wang, Lixiu Yan, Jiamin Zhang

**Affiliations:** ^1^Meat Processing Key Laboratory of Sichuan Province, College of Food and Biological Engineering, Chengdu University, Chengdu, China; ^2^College of Science and Technology, Hebei Agricultural University, Cangzhou, China; ^3^Chongqing Academy of Metrology and Quality Inspection, Chongqing, China

**Keywords:** tryptophan derivatives, 5-hydroxytryptophan, indigo, indoleacetic acid, halotryptophan

## Abstract

Tryptophan derivatives are various aromatic compounds produced in the tryptophan metabolic pathway, such as 5-hydroxytryptophan, 5-hydroxytryptamine, melatonin, 7-chloro-tryptophan, 7-bromo-tryptophan, indigo, indirubin, indole-3-acetic acid, violamycin, and dexoyviolacein. They have high added value, widely used in chemical, food, polymer and pharmaceutical industry and play an important role in treating diseases and improving life. At present, most tryptophan derivatives are synthesized by biosynthesis. The biosynthesis method is to combine metabolic engineering with synthetic biology and system biology, and use the tryptophan biosynthesis pathway of *Escherichia coli*, *Corynebacterium glutamicum* and other related microorganisms to reconstruct the artificial biosynthesis pathway, and then produce various tryptophan derivatives. In this paper, the characteristics, applications and specific biosynthetic pathways and methods of these derivatives were reviewed, and some strategies to increase the yield of derivatives and reduce the production cost on the basis of biosynthesis were introduced in order to make some contributions to the development of tryptophan derivatives biosynthesis industry.

## Introduction

The basic metabolic pathways of aromatic compound biosynthesis involve glycolytic pathway (EMP), pentose phosphate pathway (PPP), and shikimate pathway. In shikimate pathway, phosphoenolpyruvate (PEP) produced by glycolytic pathway, and D-erythrose 4-phosphate (E4P) produced by pentose phosphate pathway are used as precursors to condense to form 3-deoxy-D-arabino-heptulosonate-7-phosphate (DAHP). DAHP then undergoes a six step catalytic reaction *via* shikimate pathway to generate chorismate (Wu et al., 2021). With chorismate as precursor, chorismate is transformed into three aromatic amino acids through two ways. One way is first transformed into prephenylalanine, and then L-phenylalanine or L-tyrosine were synthesized, respectively. The other way is to generate L-tryptophan (L-Trp) from o-aminobenzoic acid (Barik, 2020; Wu et al., 2021).

Tryptophan belongs to one of the three aromatic amino acids and is the only amino acid containing an indole ring. Tryptophan not only participates in the biosynthesis and turnover of proteins and peptides, but also is absorbed into the body and transformed into a series of bioactive small multi effect compounds (Barik, 2020). It is mainly degraded through two parallel pathways, which are 5-hydroxytryptamine (5-HT) pathway and kynurenine pathway. These two pathways will produce a series of secondary metabolites. The metabolites of serotonin pathway include 5-hydroxytryptophan (5-HTP), serotonin and melatonin. The metabolites of kynurenine pathway include kynurenine and niacin. Melatonin and niacin are the final products of the above two parallel pathways, while 5-HTP, 5-HT, and inulin are intermediate metabolites (Barik, 2020; Cas et al., 2021). In addition, in plants and microorganisms, tryptophan derivatives also include chlorotryptophan, bromotryptophan (Lee and Lee, 2020), and indole alkaloids such as indole-3-acetic acid (IAA), indirubin, indigo (Xu et al., 2014).

**Figure 1:**
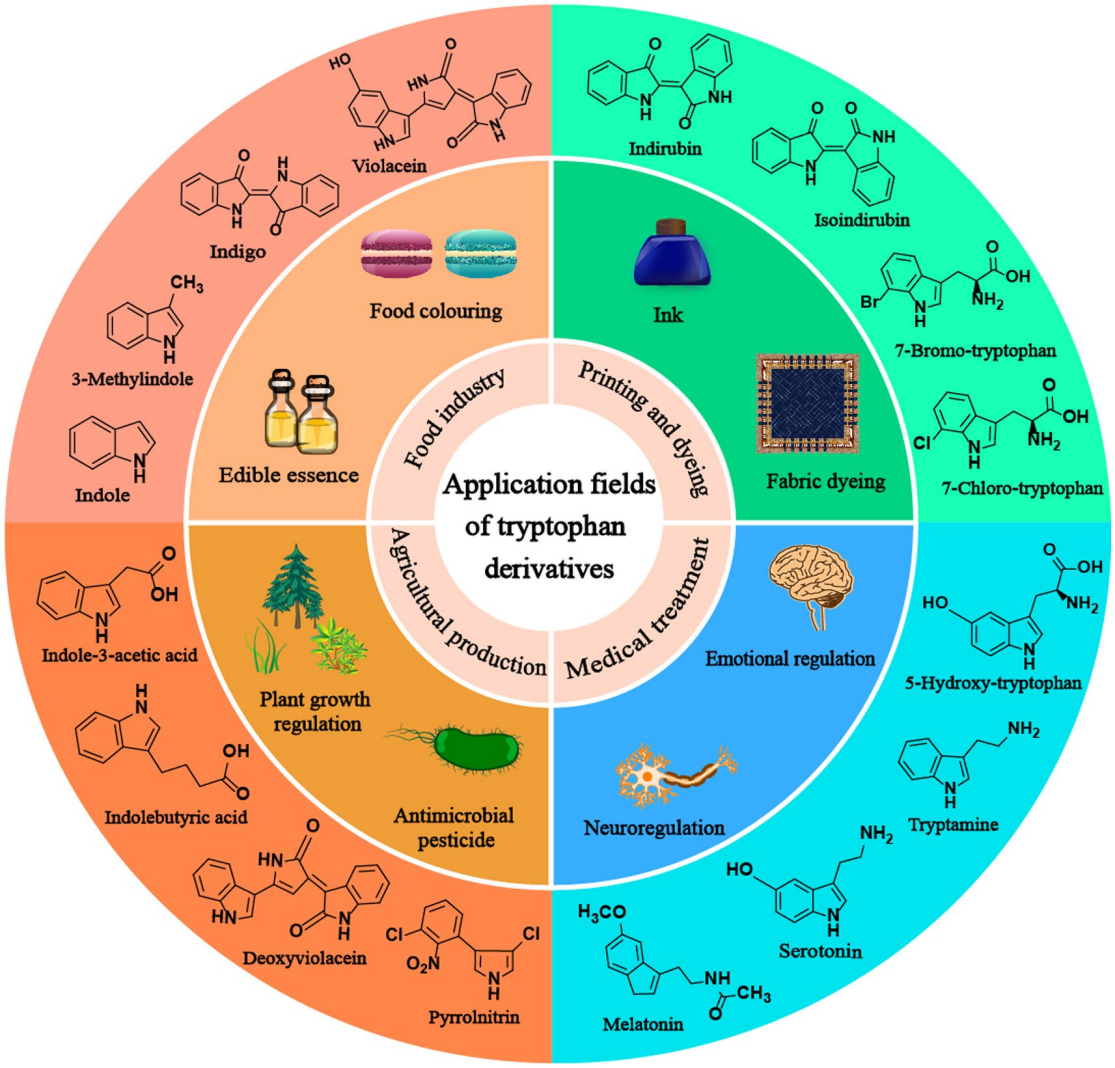
The application of tryptophan derivatives in various fields.

**Figure 2:**
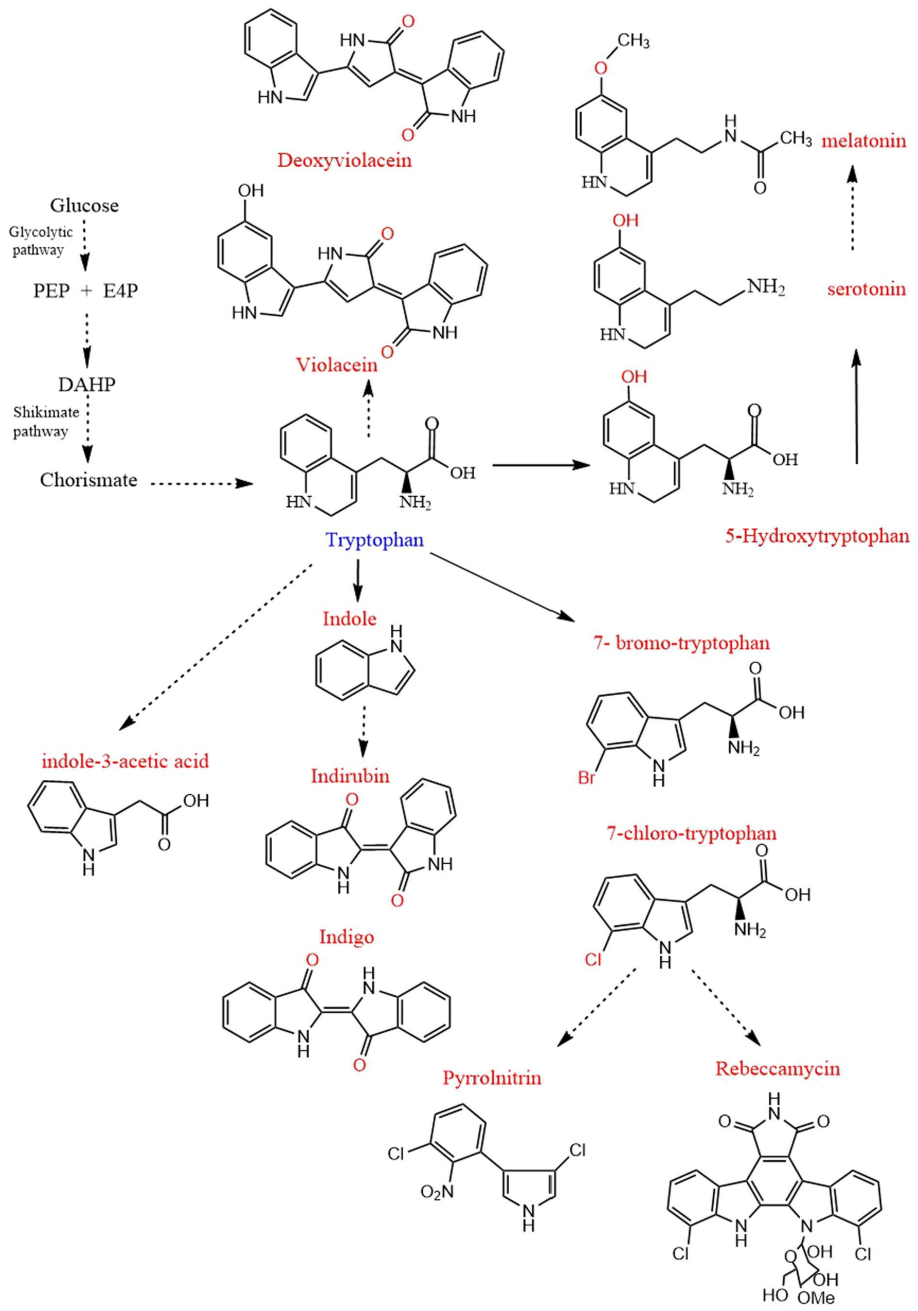
Biosynthesis pathway of tryptophan derivatives. PEP, Phosphoenolpyruvate; E4P, D-erythrose 4-phosphate; DAHP, 3-deoxy-D-arabino-heptulosonate-7-phosphate. The straight line and dotted line represent one-step and multi-step, respectively.

Tryptophan derivatives are widely needed because of their various functions, such as serotonin and melatonin, which can treat insomnia (Arnao and Hernandez-Ruiz, 2018). Halogenated tryptophan is an important intermediate or component of active substances related to the pharmaceutical, chemical and pesticide industries (Lee and Lee, 2020). Auxin (IAA) affects the root growth of plants and plays an important role in the interaction between plants and microorganisms (Leontovycova et al., 2020; Figure 1). However, due to the problems of cost, pollution and complex steps in the chemical synthesis of tryptophan derivatives, the development of modern biotechnology and synthetic biology has opened up another way for us to synthesize tryptophan derivatives (Choi et al., 2003; Lee and Lee, 2020). This paper mainly reviews the biosynthesis of common tryptophan derivatives, such as 5-HTP, serotonin, melatonin, IAA, halotryptophan, violacein, indirubin, indigo, etc. (Figure 2; Table 1).

**Table 1:** Biosynthesis of tryptophan derivatives.

Product	Host	Titer (g/L)	Time	Fermentation mode	Engineered strategy	Reference
5-HTP	*E. coli*	1.11	16 h	Batch	Expression of *phhB*, *folM*, *phhA* in BW*ΔtnaA* and QH4*ΔtnaA*	Lin et al. (2014)
Serotonin	*E. coli*	0.15	52 h	Two-step fermentation	The TrpR gene was eliminated, and Expression of PCD and DHPR genes in strain BL21(DE3)*ΔTnaa* expresses	Mora-Villalobos and Zeng (2018)
Melatonin	*E. coli*	2.0	67 h	Fed-batch	Tnaa and trpR genes were deleted and TrpH, Ddc, Aanat, Asmt genes were expressed in strain HM626	Luo et al. (2020)
Indole	*C. glutamicum*	5.7	24 h	Batch	Expression of *ectnaA*, *tnaB*, *aroP* in *C. glutamicum*	Mindt et al. (2022)
IAA	*E. coli*	3.0	24 h	Batch	Expression of *ipdC*, *aspC*, *iad1* in DH5α	Romasi and Lee (2013)
Indigo	*E. coli*	3.8	25 h	Batch	Expression of CYP102A_scat in *E. coli*	Kim et al. (2017)
Indirubin	*E. coli*	0.25	48 h	Batch	Expression of *fre*, *tnaA*, *tnaB*, *tnaAB*, *katE* and *xiaI* in BL21(DE3)	Yin et al. (2021)
Violacein	*C. glutamicum*	5.43	100 h	Fed-batch	Expression of *vioABCDE* in *C. glutamicum* 13,032	Sun et al. (2016)
Deoxyviolacein	*C. freundii*	1.9	44 h	Fed-batch	Expression of *vioABCE* in *C. freundii* (pComvio)	Jiang et al. (2012)
7-Chloro-L-tryptophan	*C. glutamicum*	0.11	24 h	Batch	Expression of *rebH* and *rebF* in *C. glutamicum* HalT2	Veldmann et al. (2019b)
7-Bromo-L-tryptophan	*C. glutamicum*	1.2	72 h	Fed-batch	Overexpression of *rebH* and *rebF* in *C. glutamicum* HalT2	Veldmann et al. (2019a)
Pyrrolnitrin	*E. coli*	/	5 days	Batch	Expression of *prnABC*D in DH5α	Liu et al. (2018)
Rebeccamycin	*Lechevalieria aerocolonigenes*	0.12	8 days	Batch	Addition of talc microparticles or glass beads to the medium to induce mechanical stress	Waliskoa et al. (2017)

## 5-HTP, serotonin, melatonin biosynthesis

**Figure 3:**
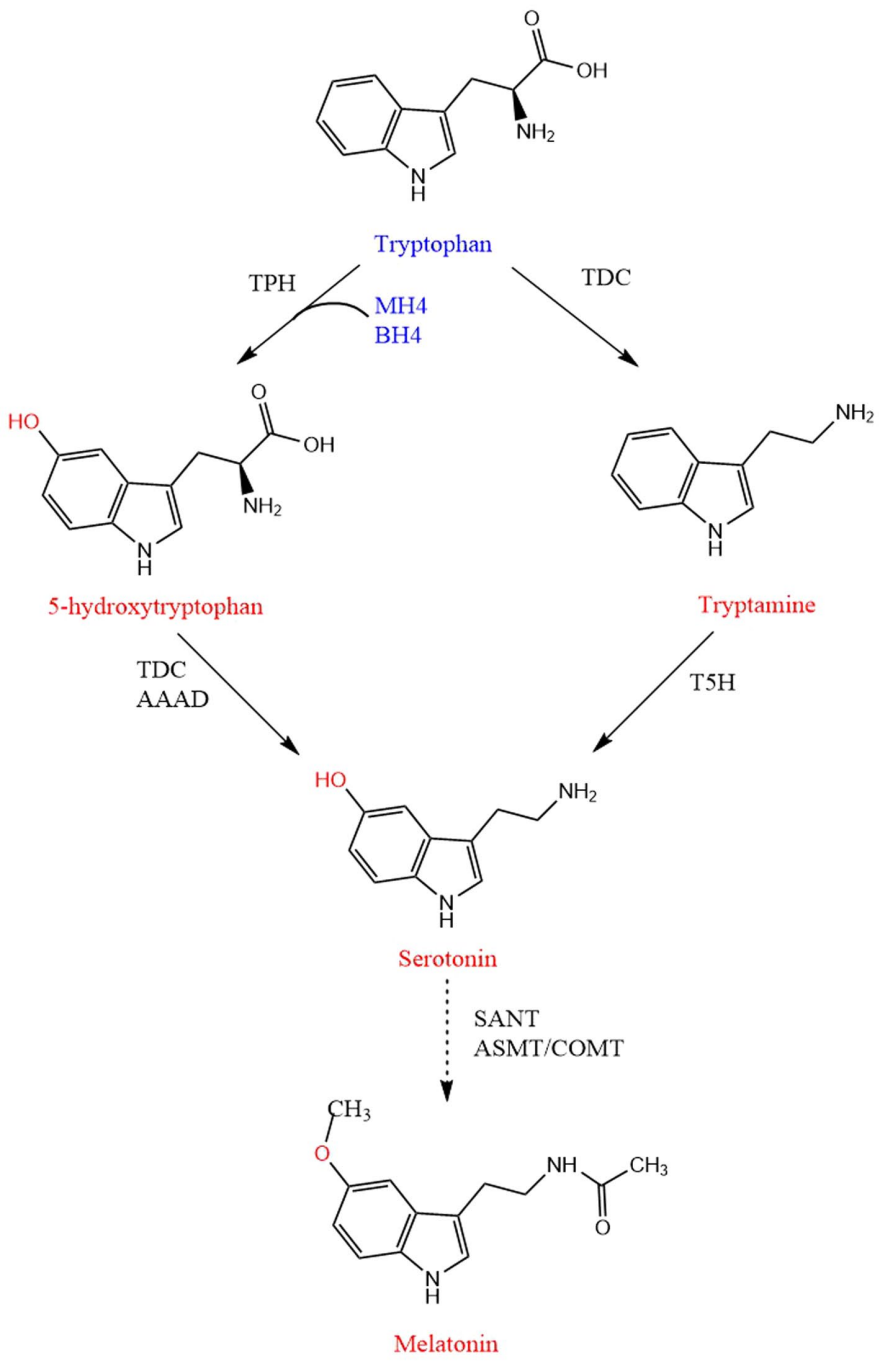
Biosynthetic pathway of 5-HTP, serotonin, and melatonin. Linear and dashed lines mean a single-step and multi-steps, respectively. TPH, Tryptophan 5-hydroxylase; TDC, Tryptophan decarboxylase; AAAD, Aromatic amino acid decarboxylase; T5H, Tryptamine 5-hydroxylase; SNAT, Serotonin N-acetyltransferase; ASMT, N-acetylserotonin O-methyltransferase; COMT, Caffeic acid O-methyltransferase; BH4, Tetrahydrobiopterin; MH4, Mtetrahydromonapterin.

5-HTP, serotonin and melatonin are products of the same tryptophan metabolic pathway (Cas et al., 2021). Tryptophan is converted to 5-HTP through tryptophan hydroxylase (TPH), and 5-HTP is converted to serotonin through aromatic acid decarboxylase. The serotonin is converted to N-acetylserotonin through arylalkylamine N-acetyltransferase, which is converted to melatonin through hydroxyindole-O-methyltransferase (Zheng et al., 2021; Figure 3).

### 5-HTP

5-HTP is a natural amino acid (AA) that does not participate in protein synthesis. It is derived from tryptophan, and the hydrogen atoms at the 5′-position on the benzene ring of tryptophan are replaced by hydroxyl groups (Liu et al., 2021). 5-HTP is the precursor of serotonin and melatonin, which can be used to treat depression, insomnia, migraine and other diseases (Wang et al., 2018). 5-HTP was originally extracted from *Griffonia simplicifolia* and other plants, but this method is expensive and raw materials are rare. Besides, the use of chemical synthesis method is cumbersome and harsh. With the progress of biotechnology, the use of microorganisms, especially *E. coli*, to synthesize 5-HTP has become the mainstream (Liu et al., 2021).

In human and mammalian cells, 5-HTP is synthesized by L-Trp hydroxylation with Fe^2+^ and BH_4_ as cofactors and O_2_ as cosubstrate catalyzed by TPH. BH_4_ is oxidized to pterin-4α-carbinolamine (BH_3_OH) during L-Trp hydroxylation and regenerated through the function of pterin-4α-carbinolamine dehydratase and dihydropteridine reductase (DHPR; Wang et al., 2018). Knight et al. found that the co-expression of the animal BH4 biosynthesis pathway and the truncated tryptamine 5-hydroxylase (T5H) from *Oryctolagus cuniculus* in *E. coli* produced 198 mg/L 5-HTP (Knight et al., 2013). However, most bacteria such as *E. coli* cannot naturally produce BH4, and they can only synthesize BH_4_ analogue tetrahydromonapterin (MH_4_; Lin et al., 2014). It is generally necessary to add exogenous BH_4_, or realize the biosynthesis and regeneration of bacterial BH_4_ through gene recombination (Germann et al., 2016).

An artificial MH_4_ recycling system was established by the expression of *phhB* from *P. aeruginosa* and *folM* [encoding dihydromonasin reductase (DHMR)] from *E. coli*. With this circulating system, *E. coli* cells could use tryptophan to produce 1114.8 mg/L 5-HTP in shake flasks (Lin et al., 2014; Figure 3). The tryptophan synthesis pathway was successfully introduced into *E. coli* to realize the *de novo* production of 5-HTP. After a series of optimization such as improving the hydroxylation activity of TPH through enzyme modification, the titer of 5-HTP was significantly increased to 1.29 g/L (Wang et al., 2018; Liu et al., 2021). Furthermore, by designing the strength of the 3-deoxy-7-phosphate synthase promoter and adjusting the copy number of the L-Trp hydroxylation plasmid, the output of 5-HTP in shake flask was increased to 1.61 g/L (Xu et al., 2020).

### Serotonin

Serotonin, also known as 5-HT, is an amino acid derivative with high added value. It can participate in emotional regulation, behavior management, and sleep cycle maintenance. It also can promote plant seed germination and growth and other physiological processes (Shen et al., 2020). Serotonin is synthesized in different ways in animals and plants. In animals, tryptophan is hydroxylated to 5-HTP through tryptophan 5-hydroxylase, and then tryptophan decarboxylase (TDC) converts 5-HTP to 5-HT, namely serotonin (Gaddum and Giarman, 1956; Cao et al., 2020). In plants, tryptophan is first converted to tryptamine by TDC, and then serotonin is produced by T5H (Goncalves et al., 2022; Figure 3).

TDC from rice was overexpressed in transgenic rice, recombinant *E. coli* (pET28b TDC), and recombinant yeast (pYES-TDC), and serotonin accumulation was detected, which confirmed that serotonin was produced under the condition of 5-HTP as substrate (Park et al., 2008). A functional T5H enzyme (GSTΔ37T5H) was constructed by a series of n-terminal deletion or labeling proteins, and then, 24 mg/L serotonin was produced by GSTΔ37T5H and TDC (Park et al., 2011). The semi rational engineering recombinant strain of aromatic amino acid hydroxylase was used to produce 5-HTP, and then the recombinant strain containing tryptophan decarboxylase was used for biotransformation of 5-HTP to produce about 154 mg/L serotonin, which was the first time to realize the production of serotonin from a simple carbon source (Mora-Villalobos and Zeng, 2018). In the first step, about 962 mg/L 5-HTP was produced by using a recombinant strain with a semi-rationally engineered aromatic amino acid hydroxylase. In the second step, biotransformation of 5HTP using recombinant strains containing TDC, about 154 mg/L of serotonin was produced. A method of producing 5-HT from tryptophan through two enzyme cascades in one pot has also been proposed (Wang et al., 2022). The tryptophan hydroxylase from *Schistosoma mansoni*, the artificial endogenous BH4 module and the dopa decarboxylase from *Harminia axyridis*, are heterologously expressed in *E. coli*. The recombinant *E. coli* can produce about 414 mg/L of 5-HT from 2 g/L of tryptophan.

### Melatonin

Melatonin, a natural product derived from tryptophan, is a major biomolecule synthesized in almost all biological organisms, including animals and plants (Back et al., 2016). Melatonin can affect circadian rhythm, mood, sleep, etc. it can also be used as a plant biological stimulant to resist biological and abiotic stress and regulate the ability of plant growth (Arnao and Hernandez-Ruiz, 2018).

Its synthesis goes through four steps. In plants, tryptophan is converted to tryptamine by TDC, and then tryptamine is converted to serotonin by T5H. Serotonin is catalyzed by serotonin N-acetyltransferase (SNAT) to complete N-acetylation, and then N-acetylserotonin is methylated by acetylserotonin methyl transferase (ASMT, a hydroxyindole-*O*-methyltransferase) to produce melatonin (Arnao and Hernandez-Ruiz, 2018). In animals, tryptophan produces 5-HTP under the combined action of TPH, cofactor BH_4_ and oxygen. Next, 5-HTP is converted to serotonin by tryptophan carboxylase. Subsequently, aralkylamine N-acetyltransferase produces N-acetyl 5-hydroxytryptamine at the expense of acetyl-CoA. Finally, N-acetyl 5-hydroxytryptamine methyltransferase is accompanied by the conversion of cofactor SAM to SAH to produce the final product melatonin (Xie et al., 2022).

In addition to animals and plants, many microorganisms can also synthesize melatonin. As seen in Figure 3, a strain of Saccharomyces cerevisiae has been cultivated, which used glucose as the only carbon source to ferment in the culture medium and produce 14.5 mg/L melatonin (Germann et al., 2016). *E. coli* can also be used for melatonin production. In several double expression box combinations, the recombinant *E. coli* expressing sheep SNAT with rice *O*-methyltransferase (COMT) produced a large amount of melatonin, which is the first report using *E. coli* to heterologously produce melatonin (Byeon and Back, 2016). Moreover, the biosynthetic pathway of melatonin was introduced into *E. coli*, and then the engineered strain produced about 2.0 g/L of melatonin through protein engineering of rate-limiting tryptophan hydroxylase, chromosomal integration of aromatic amino acid decarboxylase, and deletion of tryptophan export protein YddG (Luo et al., 2020).

## Biosynthesis of indole and its derivatives

Indole and indole alkaloids belong to the same pathway. Tryptophan is degraded into indole by tryptophanase. Indole can be converted into a variety of indole alkaloids by different enzymes through different pathways, among which IAA, indigo and indirubin are common (Chen et al., 2016).

### Indole

**Figure 4:**
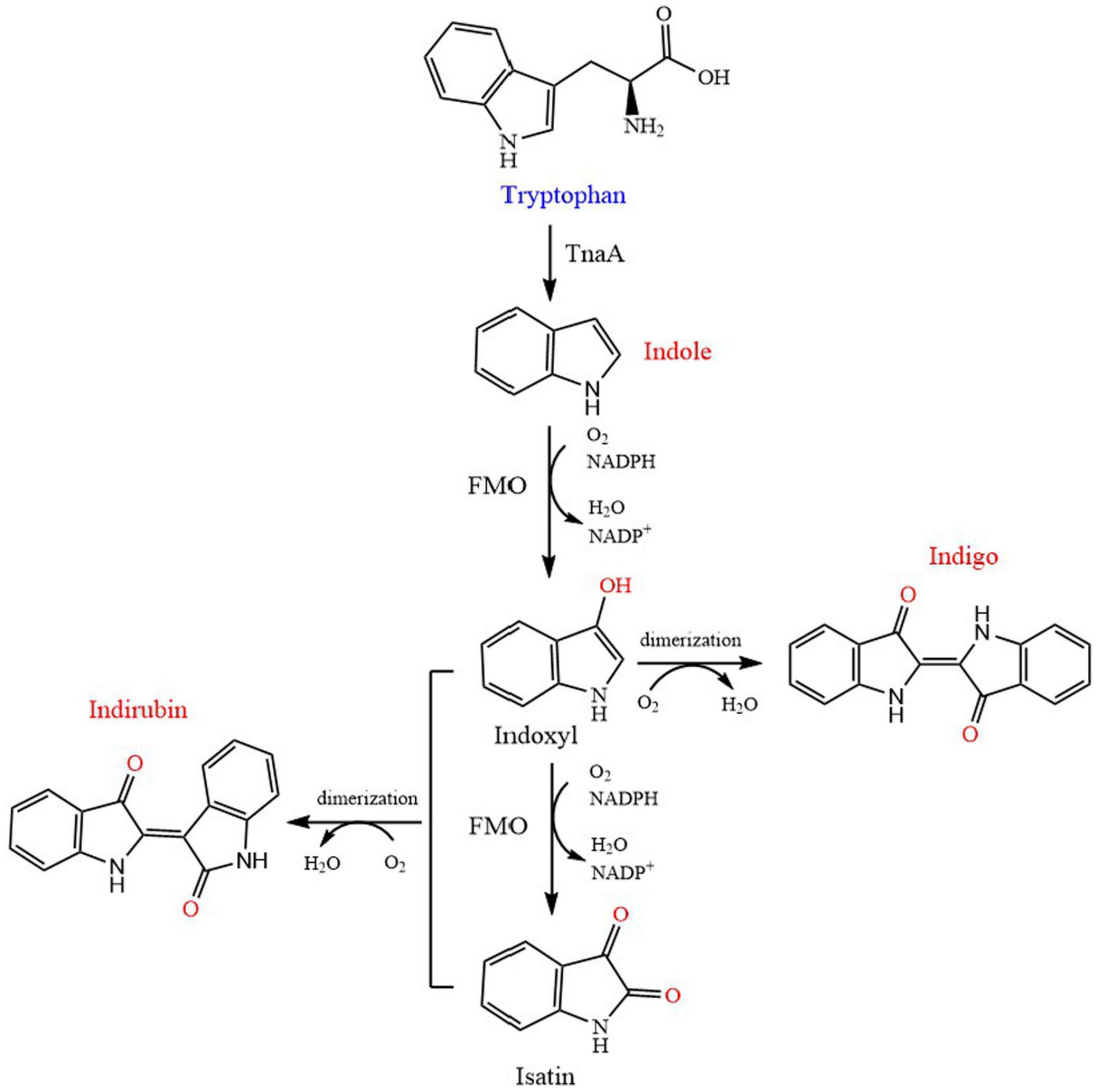
Biosynthetic pathway of indole, indigo and indirubin. TnaA, tryptophanase; FMO, flavin-containing monooxygenase.

Indole, also known as 2,3-benzopyrrole, is widely used in chemical, pharmaceutical, dye and other industries. It is an important precursor in industry, but it is also a typical nitrogen heterocyclic pollutant released into the environment (Li et al., 2020). It is a signal molecule that regulates a variety of physiological processes, including movement, biofilm formation, antibiotic resistance, plasmid stability, sustained cell formation (Han T. H. et al., 2011), indole and its derivatives strongly affect the physiological functions of bacteria and animals (Ferrer et al., 2022b). Tryptophan has been proved to be completely degraded by tryptophanase to produce indole (Watanabe and Snell, 1972; Figure 4). Li and Young (2013) reported that the final yield of indole in *E. coli* depends on the amount of exogenous tryptophan, and the transformation process was mainly dependent on the tryptophanase TnaA. On the other hand, excessive indole may also inhibit the activity of TnaA and the transport process of tryptophan.

In addition to *E. coli*, some heterologous strains can also be used to produce indole, for example, indole synthesis-related gene was introduced into *C. glutamicum*. With the expression of endogenous TSA gene or IGL gene of wheat, about 0.7 g/L indole were produced (Ferrer et al., 2022b). 5.7 g/L of indole can be produced by co-expressing the natural aromatic amino acid permease gene aroP and the tryptophanase from *Providencia rettgeri* in *C. glutamicum* (Mindt et al., 2022).

### Indole alkaloids

Indirubin and indigo belong to Indole alkaloids, which are secondary metabolites derived from plants. Many of them have important medicinal properties and have been used as drugs and dyes since ancient times (Cao et al., 2020). In addition, IAA, as plant auxin, belongs to indole alkaloids too, and IAA, indigo and indirubin belong to simple indole alkaloids (Chen et al., 2016).

#### Indole acetic acid

IAA is the most abundant auxin-active natural hormone in plants, which controls many physiological processes, such as cell proliferation and division, tissue differentiation, phototropism and geotropism reactions (Leontovycova et al., 2020). Some plant-related bacteria, fungi and yeasts, including *Agrobacterium tumefaciens*, *Azospirillum brasilense*, *Bradyrhizobium* spp. and *Enterobacter cloacae*, are known to synthesize IAA in the presence of tryptophan (Romasi and Lee, 2013).

**Figure 5:**
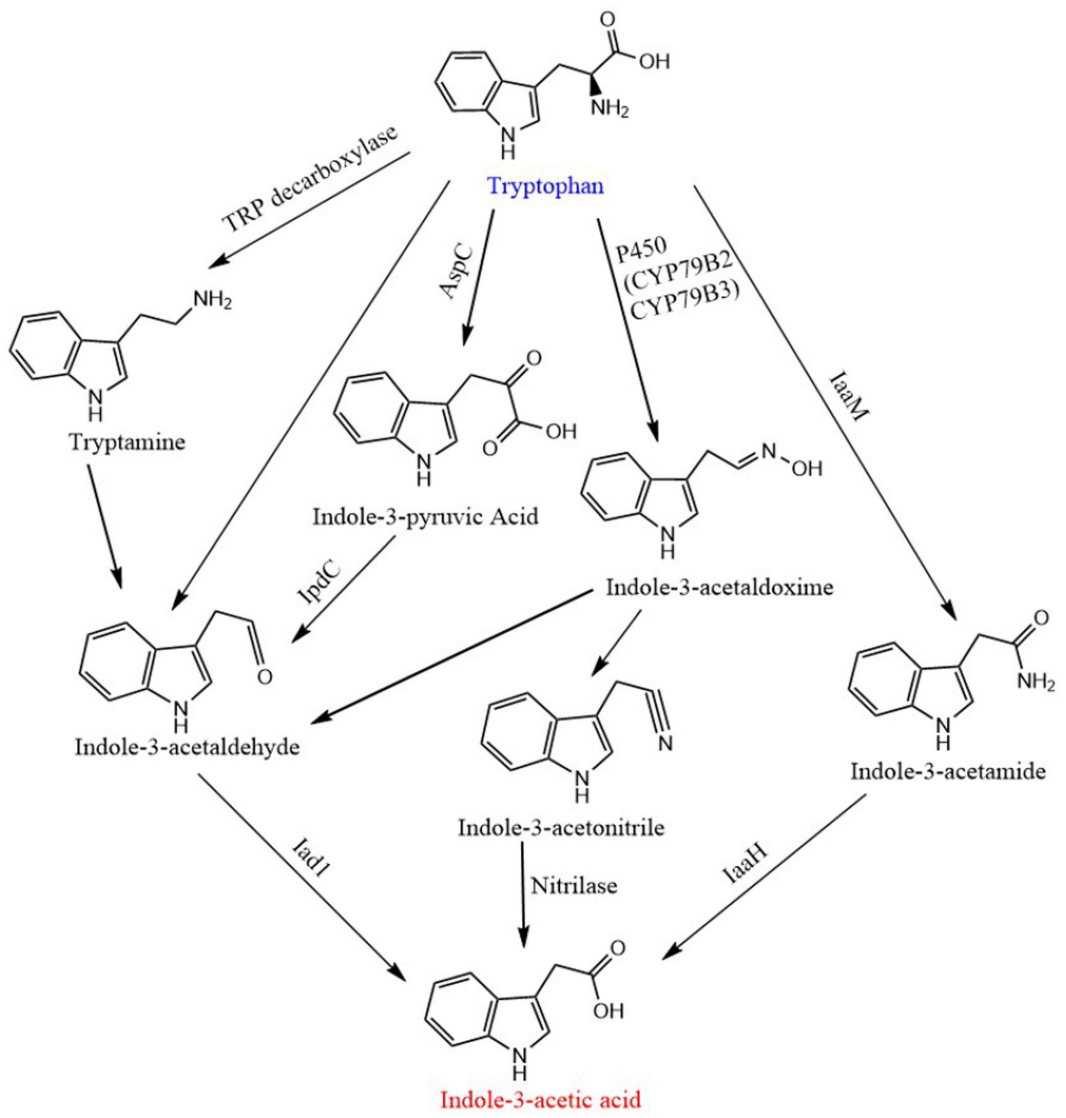
Biosynthetic pathway of in-dole-3-acetic acid. CYP79B2 and CYP79B3, cytochrome P450 monooxygenase; AapC, aminotransferase; Ipdc, indole-3-pyruvic acid decarboxylase; Iad1, indole-3-acetic acid dehydrogenase; IaaM, tryptophan 2-monooxygenase; IAAH, IAM hydrolase.

According to the main intermediates in the IAA synthesis process, the Trp-dependent biosynthesis process in plants is usually divided into four branches: indole-3-acetaldoxime (IAOx) pathway, tryptamine pathway, indole-3-acetamide (IAM) pathway and indole-3-pyruvic acid (IPA) pathway (Jiali et al., 2012). IPA pathway is the main and generally conserved biosynthetic pathway in plants, while other redundant pathways run in parallel (Casanova-Saez et al., 2021). ① IAOx pathway (also known as CYP79B pathway): Firstly, tryptophan is catalyzed by Cytochrome P450 Mono-oxygenase CYP79B2 and CYP79B3 to generate indole 3-acetaldoxime, which is then converted into indole-3-acetonitrile and indole-3-acetaldehyde (IAAld), and then IAA is generated under the catalysis of nitrilase and aldehyde oxidase, respectively. ② IPA pathway: Indole-3-pyruvate, an intermediate product, is decarboxylated to form IAAld under the action of indolepyruvate decarboxylase (IpdC), and then oxidized to IAA. ③ Tryptamine pathway: the tryptamine pathway starts with tryptophan passing through TDC catalyzes the formation of tryptamine, and then generates IAA through the intermediate product IAAld. ④ IAM pathway: The pathway consists of two distinct steps. In the first step tryptophan monooxygenase (encoded by *iaaM* gene, the gene has not been found in plants) converts tryptophan to IAM; in the second step IAM is hydrolyzed to IAA and ammonia by an IAM hydrolase (encoded by *iaaH* gene; Spaepen and Vanderleyden, 2011; Jiali et al., 2012; Romasi and Lee, 2013). The synthetic pathway of IAA in bacteria is highly similar to that in plants, except for the addition of a tryptophan side-chain oxidase pathway and it has only been demonstrated in *Pseudomonas fluorescens* CHA0 (Spaepen and Vanderleyden, 2011). In this pathway tryptophan is directly converted to IAAld bypassing IPyA, which can be oxidized to IAA (Spaepen et al., 2007). In addition, the tryptamine pathway in bacteria is opposite to that in plants. Tryptophan is first decarboxylated to tryptamine by a TDC, which is directly converted to IAAld by amine oxidase (Spaepen and Vanderleyden, 2011; Figure 5).

*E. coli* also can be used for IAA production. The ipdC (encoding indole-3-pyruvic acid decarboxylase) from *Enterobacter cloacae* ATCC 13047, aspC (encoding aminotransferase) from *E. coli* and iad1 (encoding indole-3-acetic acid dehydrogenase) from *Ustilago maydis* were cloned and expressed in *E. coli* using tac and sod promoters, and deleted a tnaA gene that mediates indole formation from tryptophan, recombinant *E. coli* produced 3.0 g/L IAA (Romasi and Lee, 2013). Similarly, The above method could also be used for *C. glutamicum*, and the recombinant strain produced 2.3 and 7.3 g/L IAA from 10 g/L L-Trp in flask culture and 5-L bioreactor, respectively (Kim et al., 2019).

#### Indigo

Indigo is a kind of blue dye which has been used for thousands of years, it is mainly used for the production of blue jeans denim (Lolita Ameria et al., 2015). It also has medicinal value, as well as hemostatic, antipyretic, anti-inflammatory and sedative properties, and can be used for anti-tumor or anti-leukemia activity (Heine et al., 2019). Indigo undergoes three stages of biosynthesis in L-Trp. First, L-Trp is decomposed into indole by tryptophanse, and then indole is oxidized to indoxyl by various oxygenase catalytic reactions, finally, indoxyl spontaneous reaction of 2 molecules generates indigo (Zhang et al., 2014; Lee and Lee, 2020; Figure 4). The representative enzymes involved in this indole oxidation reaction were naphthalene dioxygenase (NDO) and toluene dioxygenase from *Pseudomonas putida*, phenol hydroxylase from *Acinetobacter* sp. and cytochrome P450 monooxygenase from *Bacillus megaterium*，toluene monooxygenase from *Burkholderia cepacia*, flavin-containing monooxygenase (cFMO) from *C. glutamicum* and flavin-containing monooxygenase (mFMO) from *Methylophaga aminisulfidivorans* et al. (Kang and Lee, 2009; Lee and Lee, 2020).

Recombinant *E. coli* DH5α containing flavone monooxygenase (FMO) gene has been successfully cultivated, the indigo titer was 911 mg/L by batch fermentation in a 3,000 L fermenter, and the continuous fermentation in a 5 L fermenter for 110 h accumulated 23 g indigo (Han G. H. et al., 2011). In addition, a self-sufficient cytochrome P450 monooxygenase CYP102A (CYP102A_scat) cloned from *Streptomyces cattleya* was also successfully recombined in *E. coli* strain BL21(DE3), and the strain could synthesize about 1.0 g/L indigo in LB medium. This is the first self-sufficient CYP exhibiting indole hydroxylation activity to produce indigo without mutating the wild-type enzyme (Kim et al., 2017). The indole oxygenase indAB genes in *Cupriavidus* sp. SHE were also successfully cloned and heterologously expressed in *E. coli* BL21(DE3)，and the recombinant bacteria could produce 307 mg/L indigo in 1.0 g/L tryptophan medium (Dai et al., 2019).

#### Indirubin

Indirubin, a 3,2-bisindole isomer of indigo, is one of the main active ingredients of Danggui longhui Wan, which is traditionally used in China to treat chronic myeloid leukemia (Cao et al., 2020). Furthermore, indirubin and its derivatives have considerable therapeutic effects on a variety of cancers, Alzheimer ‘s disease and delayed hypersensitivity (Lee and Lee, 2020). The production of indirubin in tryptophan is the same as that in indigo. Firstly, tryptophan is oxidized to indole by tryptophanase, and then indole can be converted to 3-hydroxyindoxyl, isatin and/or 2-oxindole by heterologous oxygenases, such as NDO. Two molecules of indoxyl are spontaneously dimerized in the presence of oxygen to form indigo, whereas indoxyl and 2-oxindole/isatin are condensed to generate indirubin (Hu et al., 2010; Han et al., 2012; Zhang et al., 2014; Figure 4).

In 5 l fermentation broth containing tryptophan medium, recombinant *E. coli* DH5α cells containing FMO gene were fermented in batches to produce 5.0 mg/L indirubin. Moreover, it was found that adding 0.36 g/L cysteine to tryptophan medium could significantly increase the yield of indirubin (Han et al., 2012). There is a possible way to increase the output of indirubin. Recombinant *E. coli* expressing naphthalene dioxygenase (NDO) gene from *Comamonas* sp. MQ, induced by 2-oxindole, produced about 58 mg/L indirubin (Zhang et al., 2014). There are also some methods, such as introducing cFMO gene into *E. coli,* 103 mg/L indirubin was produced after 48 h fermentation in LB medium containing 2.5 g/L tryptophan (Lolita Ameria et al., 2015). Or introducing flavin-reducing enzyme Fre, tryptophan-lysing and -importing enzymes TnaA, TnaB and H_2_O_2_-degrading enzyme KatE, after adding 5 mmol/L tryptophan and 10 mmol/L 2-hydroxyindole, 250.7 mg/L indirubin was obtained after 48 h fermentation (Yin et al., 2021).

However, all the above methods involve the addition of tryptophan to produce indirubin, which has a high cost. Producing indirubin directly from glucose can be considered as a way to reduce production costs (Cao et al., 2020). For instance, the introduction of *Methylophaga aminothioxanthans* FMO and *E.coli* tryptophanase TnaA into *E. coli* could directly produce indirubin 0.056 g/L from glucose through fed batch fermentation (Dua et al., 2018).

## Violacein, deoxyviolacein biosynthesis

Violacein and deoxyviolacein are biindole pigments with application value of anti-bacterial, anti-virus, anti-oxidation and anti-cancer (Zhou et al., 2018). They are secondary metabolites of bacteria such as *Alteromonas luteoviolacea*, *Chromobacterium violaceum*, *Janthinobacterium lividum,* and *Pseudoalteromonas luteoviolacea* (Yang et al., 2011; Lee and Lee, 2020).

### Violacein

**Figure 6:**
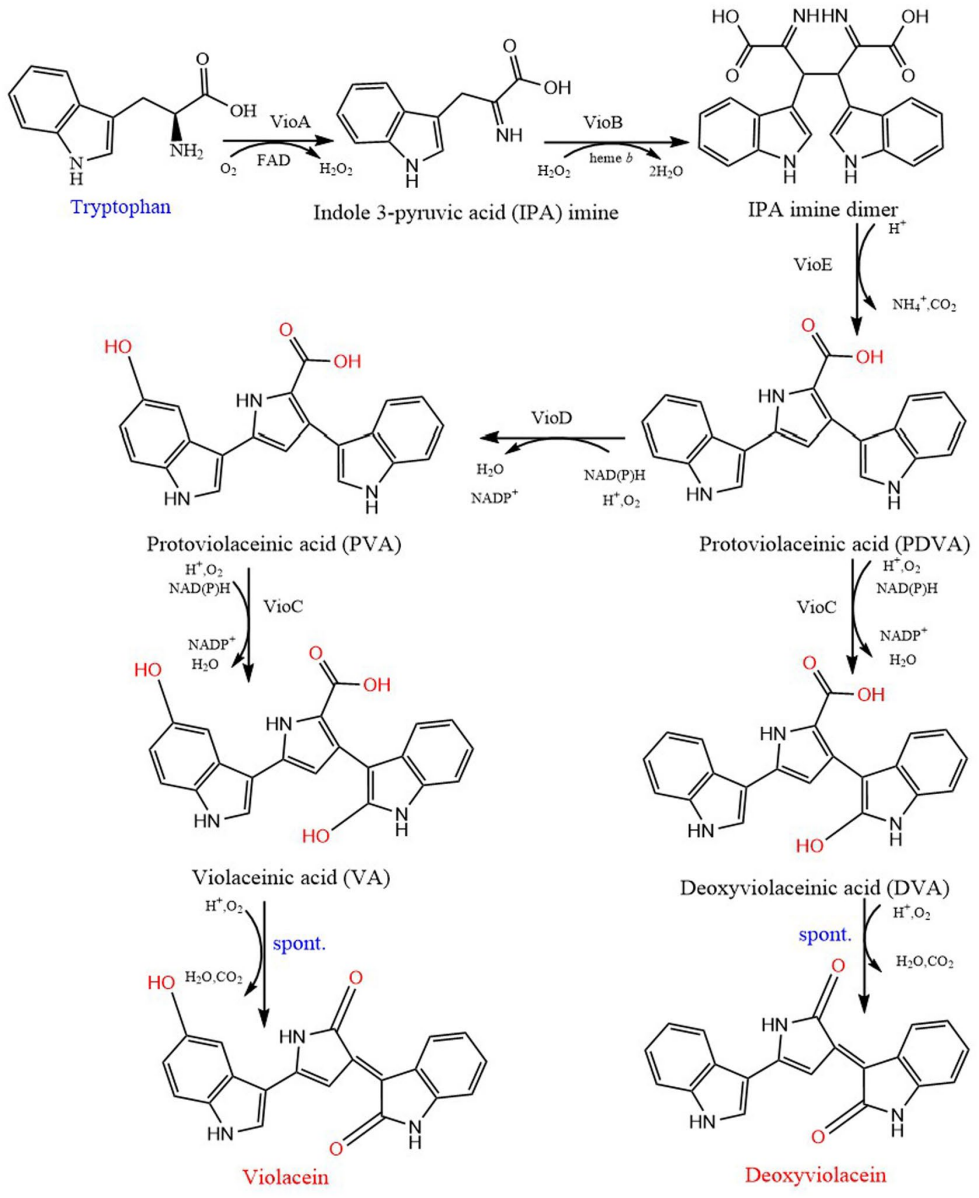
Biosynthetic pathway of violacein and deoxyviolacein. VioA, tryptophan oxidase; VioB, iminophenyl-pyruvate dimer synthase; VioE, violacein biosynthesis enzyme; VioD, protodeoxyviolaceinate monooxygenase; VioC, violacein synthase.

Violacein is a purple natural indole derivative, was first isolated from *C. violaceum*. It is synthesized by condensation of two tryptophan molecules in several bacterial genera to respond to quorum sensing signals (Ahmed et al., 2021). At first, through gene cluster separation, sequencing and heterologous expression, the production of violacein was considered to rely mainly on four adjacent genes VioA-D. Then, the fifth gene VioE was supplemented, which played an important role in the formation of violacein (Balibar and Walsh, 2006). The complete synthesis pathway of violacein was formed: VioA (flavin-dependent tryptophan-2 monooxygenase enzyme) catalyzes the oxidation of tryptophan to indole 3-pyruvic acid (IPA) imine, and reduces FAD cofactors. VioB further converts IPA into short-lived imine dimer through dimerization reaction. The imine dimer is either spontaneously converted to chromopyrrolic acid (CPA), or VioE converts the imine dimer into protodeoxyviolaceinic acid (PDVA) through the 1,2-displacement of the indole ring. PDVA is converted to protoviolaceinic acid (PVA) by adding a hydroxyl at the C5 position of an indole ring through nadp-dependent oxygenase VioD. PVA is converted into violaceinic acid (VA) by adding a hydroxyl group to the C2 position of another indole ring *via* another nadp-dependent oxygenase VioC, and then the final product violacein is generated by spontaneous oxidative decarboxylation. In addition, VioC can also use PDVA as the substrate to produce the main by-product deoxyviolacein (Ahmed et al., 2021; Park et al., 2021; Figure 6). The above five enzymes involve five coding genes vio ABCDE, and the successful expression of the operon composed of these genes requires CviI synthetase to catalyze the conversion of fatty acids or S-adenosyl methionine into AHL, which is triggered by the complex formed by AHL and CviR (a receptor; Kothari et al., 2017).

Violacein can be produced by natural production bacteria such as *Chromobacterium violaceum* (Rodrigues et al., 2012) and *Janthinobacterium lividum* (Sun et al., 2016). But the relatively low productivity of natural wild strains greatly limits the functional analysis and industrial application of violacein (Yang et al., 2011), and the violacein producing strains of *Chromo-bacterium violaceum* and *Janthinobacterium lividum* can cause rare but highly lethal infections in humans (Rodrigues et al., 2013). Therefore, the technology for heterologous expression of violacein gene cluster and production of violacein by genetic engineering has been developed and gradually matured. Pemberton et al. (1991) reported that the violacein gene cluster from *C. violaceum* was cloned and successfully expressed in *E. coli* for the first time. Later, it was reported that violacein-synthesizing gene cluster can also be heterologously expressed in *Citrobacter freundii*, the final concentration of violacein reached 4.13 g/L. This is the first report on the efficient production of violacein by genetic engineering strains in fermentation tanks (Yang et al., 2011).

In order to control the cost, increase the supply of tryptophan and improve the yield of violacein, most people began to choose the combination of the upstream pathway of tryptophan production and the downstream pathway of purple mold production (Fang et al., 2015; Park et al., 2021). By combining knockout of trpR/tnaA/pheA gene and overexpression of trpEfbr/trpD, then, the gene cluster of violacein biosynthetic pathway was introduced into the downstream of tryptophan production pathway. Recombinant *E. coli* B2/PED+pVio produced 1.75 g/L of purpomycin with glucose as the carbon source (Fang et al., 2015). After the discovery that VioE is the rate limiting step of Aspergillus purpureus synthesis, a strain of *E. coli* B8/PTRPH1-PVIo-Vioe was obtained by overexpressing VioE, 4.45 g/L violacein was obtained after fed-batch fermentation (Zhou et al., 2018). Fuethermore, the production of recombinant *E. coli* violacein could be pushed to a new level of 6.19 g/L through integrated system metabolic engineering, cell morphology engineering, inner- and outer-membrane vesicle formation, and fermentation optimization (Yang et al., 2021).

### Deoxyviolacein

Deoxyviolacein is a structural analogue of violacein and a microbial metabolite. It lacks one oxygen atom at the 6th position of indole ring (Wanga et al., 2012). It has attracted much attention due to its biological activities against tumor, Gram-positive bacteria and plant pathogenic fungi. However, the production of deoxyviolacein in wild Vio bacteria is very low, which is difficult to meet the practical needs (Andre Luis Rodrigues et al., 2014). The vioABCDE pathway was successfully expressed in *E.coli*, creating a new way for heterologous synthesis of violacein and deoxyviolacein (Pemberton et al., 1991). In addition, the production pathway of deoxyviolacein is mostly coincident with that of purplemycin. Only after PDVA is produced, VioC can directly use PDVA as the substrate to produce deoxyviolacein (Ahmed et al., 2021). Therefore, under conventional conditions, violacein produced by various bacteria is crude violacein, that is, the mixture of violacein and deoxyviolacein (Sun et al., 2016). In order to obtain pure deoxyviolacein, further purification is needed, such as silica gel (SiO_2_) column chromatography (Bilsland et al., 2018). Pathway summary shows that the expression of VioABCE without VioD would lead to a single end-product of deoxyviolacein (Park et al., 2021). Although *E. coli* with pLvioABCE (vioDdeleted Vio gene cluster) completely eliminate the production of violacein, however, the presence of a small amount of intermediate PDV associated with deoxyviolacein may regulate the violacein pathway, leading to inefficient production of deoxyviolacein (Sánchez et al., 2006).

A stable and efficient biosynthesis system for the synthesis of pure deoxyviolacein was first attempted and developed. The *vioABCE* gene cluster from *Duganella* sp.B2 was spliced and introduced into *C. freundii*, the recombinant strain produced 1.9 g/L pure deoxyviolacein in the shake flask (Jiang et al., 2012).

The synthesis of deoxyviolacein can also start directly from the synthesis of tryptophan. The araBAD promoter, which controls the expression of deoxyviolomycin cluster *vioABCE*, was deleted, deoxyviolacein biosynthesis was induced by pentose. Then, 1.6 g/L deoxyviolacein was obtained from *E. coli* dvio-8 with glycerol as the carbon source (Andre Luis Rodrigues et al., 2014). On the other hand, by integrating system metabolic engineering, cell morphology engineering, internal and external membrane vesicle formation and fermentation optimization, the yield of deoxyviolacein was further increased to 11.26 g/L (Yang et al., 2021).

## Halogenated tryptophan and its derivatives

Halogenated amino acids are widely used in pharmaceutical, chemical and agrochemical industries. They exist in a variety of natural products, including antibiotics chloramphenicol and pyrrolidomycin, plant growth regulating thienodolin and anti Eubacterium pyrrolnitrin, and rebeccamycin, which inhibits DNA topoisomerase I (Veldmann et al., 2019b). Among them, halides derived from tryptophan include 7-chloro-tryptophan and 7-bromo-tryptophan. 7-chloro-tryptophan (Karabencheva-Christova et al., 2017) is the precursor of pyrrolnitrin and rebeccamycin, 7-bromo-tryptophan (Ferrer et al., 2022a) is a precursor of the bioactive protease inhibitor TMC-95A.

### Halogenated tryptophan (7-chloro-L-tryptophan, 7-bromo-L-tryptophan)

**Figure 7:**
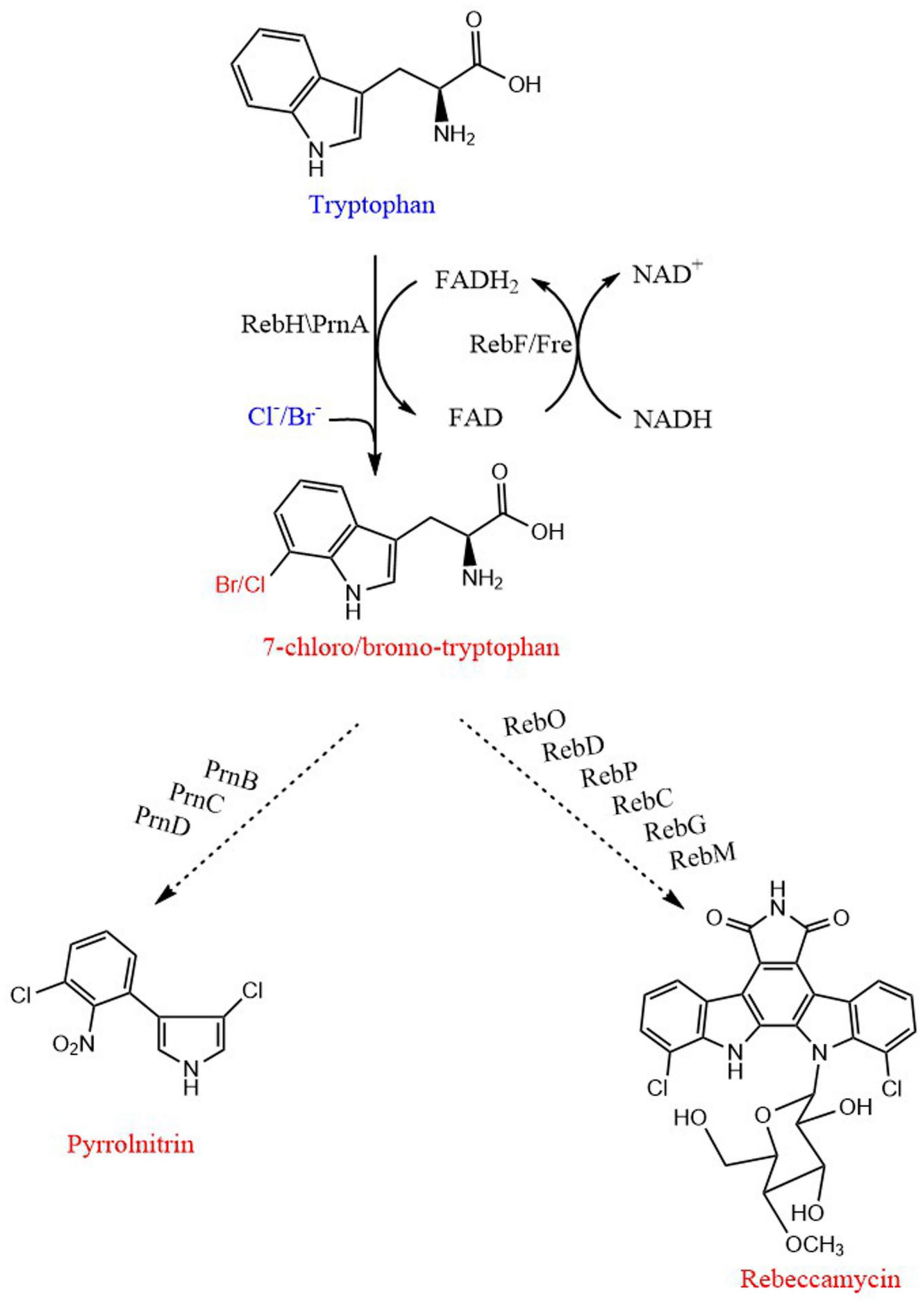
Biosynthetic pathway of 7-Halo-tryptophan, pyrrolnitrin and rebeccamycin. PrnA and RebH are FAD-dependent halogenases. RebF and Fre are NADH-dependent flavin reductases. PrnB, monodechloroaminopyrrolnitrin synthase; PrnC, monodechloroaminopyrrolnitrin halogenase; PrnD, aminopyrrolnitrin oxygenase; RebD acts as both a catalase and a CPA synthase; RebO, FAD-dependent L-tryptophan oxidase; RebP, cytochrome P450 enzyme; RebC, monooxygenase; RebG, N-glycosyltransferase; RebM, methyltransferase. The order of action of enzymes is from top to bottom. The straight line and dotted line represent one-step and multi-step, respectively.

In traditional chemical synthesis, the halogenation reaction is usually not an environmentally friendly reaction, so a green method is used by many scholars, that is, halogenase to catalyze the halogenation reaction (Phintha et al., 2021). The enzymatic catalysis of 7-halotryptophan can be completed by FADH-dependent halogenase RebH, NADH-dependent flavin reductase RebF from the biosynthesis of rebeccamycin, or tryptophan 7-halogenase PrnA and its partner flavin reductase Fre from the biosynthesis of pyrrolnitrin (Dong et al., 2005; Yeh et al., 2005; van Pee and Patallo, 2006; Figure 7). They can catalyze the regioselective chlorination/bromination of tryptophan at the 7-position of indole ring (Glenn et al., 2011; van Pee, 2012). The halogenation mechanism is that FAD, O_2_, and halogen ions are used as substrates, FAD is reduced to FADH2 by flavin reductase, combined with halogenase, and reacts with O_2_, halogen ions (Cl^−^, Br^−^) and substrates at the active site of halogenase to generate halogenated products (Dong et al., 2005; Yeh et al., 2006). For the detailed reaction principle, some scholars have proposed nucleophilic and electrophilic mechanisms respectively, but they have been proved incorrect (Chen and van Pee, 2008).

Based on the information obtained from the structural study, the reaction mechanism of tryptophan enzymatic chlorination was proposed (Karabencheva-Christova et al., 2017). In the catalytic cycle of tryptophan 7-halogenase, the reduced form of its cofactor flavin adenine dinucleotide (FAD)-FADH2 first reacts with molecular oxygen O_2_ to generate C4a-peroxyflavin, and then reacts with chlorine to generate HOCl. The HOCl formed at the FAD binding site moves through the tunnel within the enzyme and is activated to chlorinate the substrate at the tryptophan binding site. The hydrogen bond between HOCl and lysine 79 activates HOCl by increasing its electrophilicity, thereby promoting the chlorination reaction (Dong et al., 2005; Karabencheva-Christova et al., 2017).

The gram-level synthesis of halogenated tryptophan by RebH reaction has been reported, but the efficiency is too low, and it takes 8 days to complete the transformation (Frese and Sewald, 2015). Veldmann et al. (2019b) reported that a trpE gene variant encoding feedback resistant anthranilate synthase component 1, trpD encoding *E. coli* anthranilate phosphoribosyltransferase and the genes encoding RebH and RebF were overexpressed in *C. glutamicum*, could obtain 108 mg/L 7-Cl-Trp. According to the above method, with NaBr as the bromine source, the recombinant *C. glutamicum* could be used to culture in 2 L working volume to obtain 1.2 g/L 7-Br-Trp (Veldmann et al., 2019a).

In the use of enzymes, it was found that when the ratio of RebF and RebH was 3:1, the activity was the best (Yeh et al., 2005). Perhaps we can increase the production of halogenated tryptophan through this idea. In addition, tryptophan can not only be halogenated by enzyme PrnA and RebH at position 7, but also can be halogenated by tryptophan 5-halogenase (PyrH; Zhu et al., 2009) and tryptophan 6-halogenase (Thal; Moritzer et al., 2019), (SttH; Shepherd et al., 2016; Lee et al., 2021) at positions 5 and 6.

### Pyrrolnitrin

Pyrrolnitrin (3-chloro-4-(2′-nitro-3′-chlorophenyl)-pyrrole) is a tryptophan-derived secondary metabolite (Hamill et al., 1970), it was first isolated from *Burkholderia pyrrocinia* (*Pseudomonas pyrrocinia*) by Arima et al. (1964) and this compound and its derivatives can also be isolated from rhizospheric fluorescent or non-fluorescent pseudomonads, *Serratia* and *Burkholderia* (Pawar et al., 2019). Pyrrolnitrin has been used to treat skin fungal infections due to its strong antifungal activity, and has also been developed as an agricultural fungicide to inhibit soil borne fungal pathogens that affect crop yield (Kwak and Shin, 2015).

The gene cluster necessary for the synthesis of pyrrolnitrin was isolated from *Pseudomonas fluorescens* BL915, which can produce pyrrolidinitroprotein. It was composed of four genes (ORF1234), named as prnABCD, respectively (Hammer et al., 1997). Combined with the synthesis pathway of pyrrolnitrin speculated by van Pée et al. (1980), the complete gene coding catalytic synthesis pathway of pyrrolnitrin was proposed: the prnA gene product catalyzes the chlorination reaction of L-Trp to produce 7-chloro-tryptophan, and the prnB gene product catalyzes the ring rearrangement and decarboxylation to convert 7-chloro-tryptophan to monochloroaminopyrrolitrin, the prnC gene product chlorinates monodechloroaminopyrrolnitrin at the 3 position to form aminopyrrolnitrin, and the prnD gene product catalyzes the oxidation of the amino group of aminopyrrolnitrin to a nitro group to form pyrrolnitrin (Kirner et al., 1998; Figure 7).

The prnABCD operon was cloned from *plymuthica* G3 and expressed in *E. coli* DH5α, the mutant was able to overproduce pyrrolnitrin with isopropyl β-D-thiogalactoside (IPTG) induction by overexpressing prnABCD (Liu et al., 2018). In the wild-type strain, the amount of pyrrolnitrin secreted is small (Pawar et al., 2019). For example, the pyrrolnitrin production of *P. aureofaciens* ATCC 15926 strain was less than 0.3 μg/ml when grown in minimal medium, but it could be induced by N-methyl-N′-nitro-N nitrosoguanidine to increase its yield (Salcher and Lingens, 1980). In addition, the yield of pyrrolnitrin was also affected by pH, the shake flask fermentation of *P. cepacia* LT4-12-W showed that the final yield of pyrrolnitrin (168 h) almost doubled at pH 5.8 (Pawar et al., 2019). Therefore, it is necessary to fully consider the influence of various factors in the production process of pyrrolnitrin to improve the output of pyrrolnitrin as much as possible.

### Rebeccamycin

Rebeccamycin is a yellow crystalline hydrophobic substance, which was isolated from *lechevalieria aerogenes* 92 in 1985 (Nettleton et al., 1985; Bush et al., 1987). It is a halogenated natural product of indolcarbazole family, with antibiotic and anti-tumor effects. It has antibacterial activity against several Gram-positive bacteria, such as *Staphylococcus aureus* and *Streptococcus faecalis* and can also cause DNA double strand breaks and inhibit topoisomerase I, and inhibit the growth of some tumor cell lines (Sanchez et al., 2002; Waliskoa et al., 2017).

Rebeccamycin is derived from one unit of glucose, one of methionine, and two of tryptophan (Lain et al., 1990). The gene cluster of its biosynthesis was determined by Sanchez et al. (2002). Based on sequence analysis and database searches, they proposed four indole carbazole biosynthetic genes (*rebO*, *rebD*, *rebC* and *rebP*), two halogenation genes (*rebH* and *rebF*), glycosylation gene (*rebG*, renamed by ngt) and sugar methylation gene (*rebM*), as well as one regulatory gene (*rebR*) and two resistance and secretion genes (*rebU* and *rebT*), a total of 11 genes involved in rebeccamycin biosynthesis. Subsequently, mutants of the above genes were constructed, after the products of each gene were studied, through the identification of several key biosynthetic intermediates, the biosynthetic pathway of rebekamycin was found, but the RebD catalytic product was not clear (Onaka et al., 2003a). After studying the heterologous expression of RebO and RebD, a mechanism of converting IPA imines through RebO/RebD and a mechanism of generating CPA from L-Trp through two enzyme RebO/RebD system were proposed (Jones and Walsh, 2005). Furthermore, these mechanisms have been proved through experiments (Spolitak and Ballou, 2015). Therefore, the biosynthetic pathway of rebeccamycin is more complete and credible.

The biosynthesis of rebekamycin begins with L-Trp, and forms bisindole pyrrole CPA in a three-step process. The first step is the halogenation reaction of tryptophan, which is catalyzed by RebF/RebH to generate 7-chloro-tryptophan. And then, RebO, a fad-dependent L-Trp oxidase, converts 7-chloro-tryptophan to 7-chloroindole-3-pyruvic acid imine by releasing hydrogen peroxide. The oxidase RebD converts two molecules of 7-chloroindole-3-pyruvate imine to 11,11′-dichlorochromopyrrolic acid. The monooxygenase RebC and cytochrome P450 enzyme RebP perform decarboxylative ring closure. Rebeccamycin aglycon is glycosylated by RebG to 4′-o-dimethyl-rebeccamycin, and then RebM partially methylates glucose to rebeccamycin (Sanchez et al., 2002, 2006; Pommerehne et al., 2019; Figure 7).

As early as 1987, the production of rebeccamycin was attempted by using a strain with aeromycelium c-38,383-RK-2, the strain produced 663 mg/L rebeccamycin after fermentation (Bush et al., 1987). Of course, in addition to the use of wild-type bacteria producing rebekamycin, other types of bacteria can also be used to heterologously express the rebekamycin gene. For example, *S. lividans* pTOYAMAcos was used to express the whole gene cluster from *Lechevalieria aerogenes* that synthesized rebeccamycin, and the production of rebeccamycin was detected in transformed *S. lividans* (Onaka et al., 2003b). Jana et al. have reported that the enhanced culture of micro- and macroparticle has a positive effect on the production of rebeccamycin in the pellet-like morphology of bacteria particles (Waliskoa et al., 2017). In addition, Hiroyasu et al. (Onaka et al., 2015) reported that the yield of biosynthetic gene clusters of goadsporin, staurosporine and rebekamycin was significantly higher in co-culture than in pure culture. Therefore, we may improve the production of rebeccamycin by co-culturing gene clusters expressing different natural products or adding particles of different sizes in the process of culture.

## Conclusion

It can be seen from the above that tryptophan derivatives have been applied in various fields such as medicine, agriculture and life. For example, they are used to make cosmetics, textile dyeing products, drugs, pesticides, etc. And they have become an indispensable part of various fields. With the progress of metabolic engineering and synthetic biology, the chemical synthesis methods of tryptophan derivatives with pollution problems have been gradually replaced by green and healthy biosynthesis, such as the research and use of more serotonin, melatonin, indigo, indirubin and so on, they are produced by heterologous expression of genes, and their titer and efficiency are also improved. At present, the biosynthesis methods of tryptophan derivatives can be roughly divided into three categories: one is the production of tryptophan derivatives by native bacteria or plants; the second is the cultivation of mutants on the basis of native bacteria; the third is the heterologous expression of related genes. In substrate selection, tryptophan can be directly added as a substrate to generate various derivatives, and glucose, glycerol, etc. can also be used as carbon sources to integrate the tryptophan synthesis pathway and the tryptophan derivative pathway in the same strain to achieve *de novo* synthesis.

In the process of biosynthesis of tryptophan derivatives, high yield has always been the pursuit of everyone. For how to improve the yield, the methods of various derivatives described above are different. Here, the author summarizes these methods: the first is to control the proportion of various enzymes. The appropriate proportion of enzymes can maximize the synthesis of catalytic products. Second, the substrate concentration should be controlled. High substrate concentration would inhibit the biological activity and reduce the productivity of the final product. The third is to inhibit the consumption of the final product, when the bacteria produce tryptophan derivatives, the bacteria will also use the final product to meet their own life activities. The fourth is the combined culture of bacteria, there is a mutual promotion between the enzymes and their catalytic products in several bacteria, the combined culture of multiple colonies is expected to increase the yield, but further purification may be needed when various products are harvested. In addition, there is also a complex relationship between cell morphology and yield. Culture parameters including inoculum size and age, genetic factors, culture medium composition, pH, mechanical stress, mass transfer, viscosity, osmotic pressure, solid particles, addition of polymers, surfactants or chelates, temperature, and the geometry of reactors and agitators can significantly affect their morphology, which in turn affects yield.

The biosynthesis of tryptophan derivatives is indeed a green and safe way than chemical synthesis. However, the cost of using tryptophan as the substrate is higher. Therefore, it is a major trend to synthesize tryptophan derivatives from the source using glucose, glycerol and other carbon sources. However, the yield of synthesize tryptophan derivatives from glucose is low. It is also because of this problem that many tryptophan derivatives, such as pyrrolnitrin, have not yet met the requirements of industrial biological manufacturing. According to the above description, we can try to improve the enzyme activity and optimize the culture conditions to improve the yield. On this basis, we can also try to cultivate more suitable strains or use genetic engineering to optimize the synthesis pathway to achieve efficient synthesis of tryptophan derivatives from the source. Finally, because of the wide variety of secondary metabolites of tryptophan, there are still blank in the function, synthesis pathway, and relationship between many secondary metabolites. The tryptophan derivative system needs to be further expanded and improved, and they are also worth exploring for more efficacy and more functions in various fields.

## Author contributions

SX, ZW, BW, BH, TB, YZ, and WW drafted the manuscript. JC, LY, and JZ finalized the manuscript. All authors contributed to the article and approved the submitted version.

## Funding

This work was supported by the National Natural Science Foundation of China (22108017), the Natural Science Foundation of Sichuan Province (2022NSFSC1614), the Starting Grant from Hebei Agricultural University, China (YJ201950) to ZW, the Key Research and Development Program Projects of Hebei Province (22322905D), and the Open Funding Project of Meat Processing Key Laboratory of Sichuan Province (22-R-11 and 22-R-24).

## Conflict of interest

The authors declare that the research was conducted in the absence of any commercial or financial relationships that could be construed as a potential conflict of interest.

## Publisher’s note

All claims expressed in this article are solely those of the authors and do not necessarily represent those of their affiliated organizations, or those of the publisher, the editors and the reviewers. Any product that may be evaluated in this article, or claim that may be made by its manufacturer, is not guaranteed or endorsed by the publisher.
